# SCIG administration: A promising and patient convenient alternative for those receiving long-term IVIG

**DOI:** 10.5339/qmj.2022.fqac.29

**Published:** 2022-03-28

**Authors:** Slim Hassini, Soosan Samuel, Sibi Shibul, Maryam Al-Nesf

**Affiliations:** ^1^Allergy and Immunology Division, Department of Medicine, Hamad Medical Corporation, Doha, Qatar E-mail: shassini@hamad.qa

**Keywords:** IVIG, PID, SCIG

## Abstract

Background: Intravenous immunoglobulin (IVIG) therapy has been used as antibody replacement therapy in primary immunodeficiency diseases (PID) for more than 50 years. In this study, we aimed to define IVIG usage and adverse reactions and complications in PID and explain how subcutaneous immunoglobulin (SCIG) replacement therapy is an alternative that improves the patient experience. In addition, the additional nursing responsibilities associated with this service were also identified.

Methods: Data and service satisfaction surveys for the last 10 years were reviewed from the Allergy and Immunology Division log registry for those on IVIG and SCIG.

Results: IVIG practice: Most patients currently on IVIG in our unit have PID. Adverse reactions occur during the initial 30 to 60 minutes of the infusion and are mild and self-limited. Infusion reactions are more likely to occur in patients receiving IVIG for the first time. Infusion-related complications included pyrogenic reactions, allergic reactions, and vasomotor symptoms. Complications reported in the literature such as the transmission of blood-borne pathogens and other serious complications, including thrombotic events, renal adverse events, and aseptic meningitis were never reported. Pyrogenic reactions occurred at a rate ≥ 100 mL/hr in at least 3 patients, and a slower infusion rate of ≤ 75 mL/hr mitigated this rate-related complication.

SCIG program: This program started in Qatar in 2017. Usually, the clinician assesses and evaluates several factors to help select candidates for this therapy, including the perceptions of inconvenience and/or pain of IV infusions, presence of difficult vein access, and other relevant clinical and social factors. Once training in the appropriate techniques has been accomplished (3–6 sessions), it is most often self-administered in the home setting by the patient or a parent for a child. Table 1 summarizes patients on SCIG.

Additional nursing responsibilities: The nursing role in subcutaneous IgG administration is primarily that of an educator and to help the patient/family become independent. This can be achieved by the assessment of appropriate patient selection for self-administration. Determining which patients are suitable include adequate patient education with return demonstration of the necessary skill set, monitoring parameters, educating patients about their medication, and providing educational resources and support.

Conclusion: SCIG administration can be a convenient alternative for patients with PID receiving long-term IVIG.

## Figures and Tables

**Table 1:** Demographic and clinical characteristics of patients with PID on SCIG

Demographic & Clinical Characteristics of pts receiving SCIG (Hizentra) In Qatar							

Patient	Age (years)	Age at first symptoms (years)	Diagnosis	Dose (grams)	Frequency	Discontinued?	Reasons for discontinuation

**Pt 1**	24	18	SAD	12 g	weekly	No	

**Pt 2**	29	15	CVID	10 g	weekly	No	

**Pt 3**	55	24	CVID	8 g	weekly	No	

**Pt 4**	30	10	CVID	8 g	Once in 6 days	No	

**Pt 5**	37	18	CVID	12 g	weekly	No	

**Pt 6**	19	10	XLA	20 g	weekly	No	

**Pt 7**	21	12	PIDD	8 g	weekly	No	

**Pt 8**	31	18	CVID	12g	weekly	No	

**Pt 9**	48	32	SAD	8 g	Once in 5 days	No	

**Pt 10**	51	38	SAD	8 g	weekly	No	

**Pt 11**	40	34	PIDD	8 g	weekly	No	

**Pt 12**	16	10	PIDD	25 g	q.4 weeks	No	

**Pt 13**	28	14	CVID	30 g	q.4 weeks	No	

**Pt 14**	53	36	SAD	12 g	weekly	No	

**Pt 15**	43	21	2ry IDD	20 g	Once in 10 days	No	

**Pt 16**	30	18	CVID	12 g	Once in 5 days	yes	swelling and subcutaneous nodules

**Pt 17**	49	32	SAD	8 g	Once in 6 days	yes	swelling and redness in the infusion site

Demographic and clinical characteristics of patients receiving SCIG (HyQvia) in Qatar							

**Pt 18**	22	13	XLA	20 g	q.3 weeks	yes	local infection

**Pt 19**	52	39	SAD	30 g	q.2 weeks	yes	unavailability of SCIG

**Pt 20**	37	19	XLA	40 g	q.4 weeks	yes	unavailability of SCIG

**Pt 21**	30	12	PIDD	60 g	q.4 weeks	yes	unavailability of SCIG


